# The Effects of Simulated Wildland Firefighting Tasks on Core Temperature and Cognitive Function under Very Hot Conditions

**DOI:** 10.3389/fphys.2017.00815

**Published:** 2017-10-24

**Authors:** F. Michael Williams-Bell, Brad Aisbett, Bernadette A. Murphy, Brianna Larsen

**Affiliations:** ^1^School of Health and Community Services, Durham College, Oshawa, ON, Canada; ^2^Faculty of Science, University of Ontario Institute of Technology, Oshawa, ON, Canada; ^3^Institute for Physical Activity and Nutrition (IPAN), School of Exercise and Nutrition Sciences, Deakin University, Geelong, VIC, Australia; ^4^Faculty of Health Sciences, University of Ontario Institute of Technology, Oshawa, ON, Canada; ^5^School of Exercise and Nutrition Sciences, Deakin University, Burwood, VIC, Australia; ^6^Griffith Sports Physiology, School of Allied Health Sciences, Griffith University, Gold Coast, QLD, Australia

**Keywords:** cognition, thermoregulation, heat stress, firefighters, occupation

## Abstract

**Background:** The severity of wildland fires is increasing due to continually hotter and drier summers. Firefighters are required to make life altering decisions on the fireground, which requires analytical thinking, problem solving, and situational awareness. This study aimed to determine the effects of very hot (45°C; HOT) conditions on cognitive function following periods of simulated wildfire suppression work when compared to a temperate environment (18°C; CON).

**Methods:** Ten male volunteer firefighters intermittently performed a simulated fireground task for 3 h in both the CON and HOT environments, with cognitive function tests (paired associates learning and spatial span) assessed at baseline (cog 1) and during the final 20-min of each hour (cog 2, 3, and 4). Reaction time was also assessed at cog 1 and cog 4. Pre- and post- body mass were recorded, and core and skin temperature were measured continuously throughout the protocol.

**Results:** There were no differences between the CON and HOT trials for any of the cognitive assessments, regardless of complexity. While core temperature reached 38.7°C in the HOT (compared to only 37.5°C in the CON; *p* < 0.01), core temperature declined during the cognitive assessments in both conditions (at a rate of −0.15 ± 0.20°C·hr^−1^ and −0.63 ± 0.12°C·hr^−1^ in the HOT and CON trial respectively). Firefighters also maintained their pre-exercise body mass in both conditions, indicating euhydration.

**Conclusions:** It is likely that this maintenance of euhydration and the relative drop in core temperature experienced between physical work bouts was responsible for the preservation of firefighters' cognitive function in the present study.

## Introduction

Wildland fires destroy vast amounts of land on a global scale (Liu et al., 2010) and represent a significant financial burden for both individuals and government (Hyde et al., 2007). Such fire events are becoming more severe (Hennessy and Wales, 2005), likely due to the increasingly hot and dry summers experienced as a result of climate change (Westerling et al., 2006) as well as continued development and urban sprawl (Buxton et al., 2011). With increased fire severity comes increased demands placed on wildland firefighters, which may exacerbate the risks already associated with the occupation (Aisbett et al., 2012).

Firefighters are often required to make life altering decisions under conditions of extreme physical and psychological stress (Morley et al., 2012; Robinson et al., 2013). Previous research has identified that wildland fire suppression challenges firefighters' decision making ability, analytical thinking, problem solving, and situational awareness (Hayes et al., 2013). Moreover, interviews conducted after a fire event have shown that firefighters often underestimate their cognitive workload, which can impair their decision making on the fireground (Elliott et al., 2009). In the field, firefighters are challenged not only with making critical decisions, but doing so at the same time as performing physical work in hot ambient temperatures (Hancock and Vasmatzidis, 2003). Moreover, firefighters performing occupational operations are required to wear thermal protective clothing (TPC) that can impair normal thermoregulation (Morley et al., 2012). This impairment could induce hypohydration and hyperthermia, both of which can negatively impact cognitive function (Morley et al., 2012).

The majority of research investigating heat exposure and cognitive function during physical work (or exercise) has implemented continuous experimental protocols with short or no rest periods (e.g., Sharma et al., 1986; Rayson et al., 2005; Radakovic et al., 2007; Morley et al., 2012). Data reported in the literature is currently equivocal; some studies have reported no change in cognitive function immediately following exercise in 33–35°C (Morley et al., 2012), and no change in rapid visual information processing, reaction time, and spatial memory span 30 min following simulated firefighting (Rayson et al., 2005). Alternatively, impairments in psychomotor vigilance and recall have been reported 1 h following exercise and post-recovery rehydration (Morley et al., 2012). Robinson et al. (2013) also reported a decline in visual declarative memory immediately following a simulated bout of shipboard firefighting in a ship galley heated to between 60 and 130°C, as well as impairments in working memory 20-min post-simulation. Furthermore, a decrease in mental alertness, associative learning, and reasoning after a stepping exercise (Sharma et al., 1986), as well as impaired working memory during passive hyperthermia (Racinais et al., 2008), has been observed in extreme heat conditions (45–50°C). On balance, it appears that when no decrement was observed it was due to a dissipation of the thermal stress (Rayson et al., 2005) or the simplicity of the cognitive tasks (Radakovic et al., 2007). Alternatively, impairments in cognitive function appear to occur during more complex tasks (Radakovic et al., 2007; Morley et al., 2012) or as a result of dehydration (Sharma et al., 1986).

There are still numerous unanswered questions surrounding the effects of heat exposure on cognitive function in firefighters. Based on the various methodologies utilized in the literature to induce heat stress and assess cognitive function, it is currently difficult to predict how hot ambient temperatures will impact wildland firefighters' cognitive function when performing work tasks that more accurately simulate wildfire suppression activities. Therefore, the purpose of this study was to quantify the effects of very hot (45°C) ambient conditions on cognitive function following periods of intermittent simulated wildfire work, when compared to temperate conditions (18°C). These conditions were chosen because, although job tasks can occur in substantially hotter conditions, the majority of wildland firefighting activities are performed away from the radiant heat while exposed primarily to the ambient conditions (Karter, 2012; Fahy et al., 2014). Thus, the current study utilized 45°C in the heat trial to replicate the more “extreme” ambient temperatures faced by wildland firefighters in Southern Australia (Teague et al., 2010). The outcomes of this study will provide fire agencies with critical information on the likely magnitude of any cognitive impairment experienced during extreme heat exposure, so additional safety measures can be implemented as necessary.

## Materials and methods

### Participants

Ten healthy male volunteer firefighters were recruited from the Country Fire Authority (CFA; Victoria, Australia). Participants completed a medical questionnaire prior to commencement of the study to ensure they were physically able to perform the work protocol. Ethical approval was obtained from the Deakin University Human Ethics Committee, and written informed consent was attained prior to each subjects' first visit to the laboratory. Age, height (stadiometer; Fitness Assist, England), and semi-nude (i.e., underwear only) body mass (Tanita, USA) were recorded prior to testing. Firefighters ranged from 19 to 61 years old (mean: 41 ± 5 years) with 2–40 years of service (mean: 12 ± 4 years) in the CFA. The average height of the firefighters was 1.80 ± 0.03 m, body mass was 89.2 ± 2.8 kg, and BMI was 27.5 ± 1.0 kg^.^m^−2^. Following the experimental protocol, semi-nude body mass was re-measured to determine any exercise-induced dehydration. In all trials, participants wore their own firefighting TPC (weighing ~5 kg) consisting of a two-piece jacket and pants set made from Proban® cotton fabric (Protex®, Australia), suspenders, boots, gloves, and helmet. It should be noted that a portion of the data collected in the broader study (e.g., *ad libitum* fluid intake, heart rate data) has been published previously (Larsen et al., 2015).

### Experimental protocol

Participants performed a simulated fireground task in very hot and dry (HOT, mean ± SD; 45.0 ± 0.3°C, 26.9 ± 2.0% humidity) conditions, as well as a temperate control trial (CON, mean ± SD; 18.0 ± 0.0°C, 55.7 ± 1.2% humidity). The trials were counterbalanced to minimize carryover effects, and separated by at least 1 week to allow for full recovery and reduce any potential for heat acclimation. A light wind was generated via the use of a pedestal fan situated in the corner of the environmental chamber (Sunbeam, Botany NSW, Australia). Wind speed was measured at four points within the climate chamber (Voitsch, Germany) and was maintained at an average of <1.0 m^.^s^−1^. Participants were familiarized with the physical task in a separate session within a week of testing (in an environment identical to the CON trial) in order to minimize any potential learning effect (Hopkins et al., 2001) confounding the experimental trials. Environmental temperature and humidity were measured by the climate chamber and recorded in 10-min intervals.

Participants were required to perform 3 h of intermittent, simulated rakehoe work interspersed with a low-intensity stepping test (to simulate walking on the wildland fire ground) (Budd et al., 1997). A 3-h protocol was selected to simulate key job tasks during wildfire suppression, while also reducing the risks associated with performing prolonged physical work in very hot conditions. Specific end-point criteria for terminating the work sessions were: (i) core temperature reaching 39.5°C, heart rate reaching or exceeding 100% of age-predicted maximum for two consecutive minutes, or if the participant experienced symptoms of heat illness (e.g., dizziness, nausea), exhaustion, or volitional fatigue (Selkirk and McLellan, 2004).

In the 24 h prior to testing, participants were asked to document their activities (e.g., diet, sleep and exercise behaviors) and were asked to maintain a similar routine prior to each session. Participants were asked to refrain from excessive sun exposure, alcohol, and hard exercise, maintain adequate hydration, and ensure adequate sleep the night prior to testing in order to minimize the risk of heat illness (Armstrong et al., 2007). Experimental testing was conducted during the autumn and winter months (May through July) to minimize the potentially confounding impact of heat acclimatization. Approximately 8 h prior to testing, participants ingested a core temperature capsule (Jonah, Minimitter, Oregon) in order to allow adequate time for the capsule to transit from the stomach to the small intestines (Lee et al., 2000).

In order to minimize the occurrence of hypohydration, participants were told to slowly drink beverages (~5–7 mL^.^kg^−1^) at least 4 h prior to testing (Sawka et al., 2007), in accordance with American College of Sports Medicine (ACSM) guidelines. Upon arrival, participants completed an initial cognitive baseline assessment followed by instrumentation. Participants then dressed in their firefighting TPC and completed a second cognitive baseline assessment within the climate chamber condition. Participants were allowed to drink cool water (mean temperature range 13.8–15.9°C) *ad libitum* throughout testing.

### Rakehoe task

This task simulates building a firebreak using a rakehoe, and was chosen due to its importance in both land management and tanker-based wildland firefighting (Budd et al., 1997; Budd, 2001; Phillips et al., 2012). This activity has been described as intense (86.2 ± 10.8% HR_max_), with the average duration of a single raking task bout previously shown to be 38 s (ranging from 1 to 127 s; Budd et al., 1997). The task simulation involved raking 29 kg of material (large tire crumb, small tire crumb and ropes to simulate leaves, branches and debris) from one end of a rectangular (2 × 0.9 m) wooden box to the other (considered to be one repetition), using a rakehoe (i.e., a specialized combination rake and hoe). Repetitions were measured to the nearest quarter, and the same researcher counted repetitions for each participant to ensure a consistent standard.

### Step test

The present research used a modified version of a sub-maximal step test (Siconolfi et al., 1985) to simulate the light intensity activity (e.g., walking) performed on the fireground (Aisbett and Nichols, 2007; Raines et al., 2012, 2013). Only the lowest intensity (estimated at 4 METs) phase of the test was utilized, consistent with the energy expenditure of “walking with purpose” (Powers and Howley, 2007) and similar to the energy expenditure reported for non-rake activities on the fireground (Budd et al., 1997). The test comprised stepping up and down on a 25-cm platform, at a rate of 17 steps^.^min^−1^ (as timed by a metronome; Siconolfi et al., 1985), for 8-min.

### Work to rest ratios

Over the course of a fire ground work shift, wildland firefighters have been observed to have periods of predominantly sedentary activity (51–66% of a given time period) interspersed with brief bouts of moderate/vigorous activity (Cuddy et al., 2007; Raines et al., 2012, 2013). In order to simulate the intermittent nature of firefighting work (Aisbett and Nichols, 2007; Cuddy et al., 2007; Rodríguez-Marroyo et al., 2011), the 3-h protocol was broken up into six 30-min bouts, during which time participants spent 1-min on the rakehoe task followed by 8-min on the stepping task, followed by a second 1-min rakehoe bout with a subsequent 20-min rest period (total time: 30-min). This circuit was performed six times over the 3-h testing period. The rest periods equated to spending 67% in the sedentary range, which is similar to the upper limit observed during fire suppression work (Cuddy et al., 2007; Raines et al., 2012, 2013). In both trials, participants were allowed to remove their helmet and open their jacket during the 20-min rest periods, as would be typical of firefighters during rest/recovery periods on the fireground (Raines et al., 2012, 2013).

### Cognitive function measurements

The Cambridge Neuropsychological Test Automated Battery (CANTAB) cognitive testing battery (Cambridge Cognition Ltd., Cambridge, UK) comprises three batteries, each addressing a specific area of cognition. Based on the key competencies (decision making ability, analytical thinking and problem solving, and situational awareness) identified by Hayes et al. (2013), three individual tests were carefully selected from the CANTAB batteries: (i) PAL (paired associates learning) and (ii) RTI (reaction time) tests (Robbins et al., 1994), and (iii) a SSP (spatial span) test (Owen et al., 1990). The tests were administered using a computer with a touch-sensitive screen with an administrator present to describe and run the battery. These tests have been previously shown to avoid ceiling (Coull et al., 1995) and floor (Sahakian et al., 1988) effects. The touch-screen computer provided detailed data recording and analysis of accuracy and speed.

To avoid learning effects, each participant performed the cognitive testing battery twice (in a temperate environment of 22°C) before and after familiarization of the physical task in the CON environment. Prior to the experimental protocol, a double baseline (first baseline in 22°C, second baseline in the experimental temperature and humidity) was conducted to further minimize learning effects. The second baseline was used as cog 1 to ensure that all cognitive data was collected at the same temperature and humidity within each condition. During the experimental protocol, the PAL and SSP tests were assessed before (cog 1) and during the last 20-min of each hour in the 3-h protocol (i.e., 0:40, 1:40, 2:40) for cog 2, 3, and 4, respectively. In order to ensure the cognitive testing battery performed throughout the exercise trial remained under 20 min (the length of the rest periods throughout the protocol), RT1 was only tested pre-and post-exercise (i.e., cog 1 and cog 4).

#### Reaction time (RTI)

The RTI test (Morris et al., 1986) is a latency task where the subject is asked to touch a spot, contained in a circle, as fast as possible immediately after it appears onscreen. The five-choice reaction time test shows five circles and the subject is asked to touch a spot in one of the five circles immediately after it appears. This task uses a procedure to separate response latency from movement time, as well as recording accuracy (Robbins et al., 1994). Five-choice reaction time, when taken together with five-choice movement time, provides the opportunity to separate out any speeding or slowing of motor function from any speeding or slowing of cognitive function (Gooday et al., 1995; Riekkinen et al., 1998).

#### Paired associates learning—PAL

The PAL test (Sahakian et al., 1988) evaluates episodic and visual memory, but also relies on the individual's ability for spatial planning. The participants have to remember the location of different patterns appearing on the screen (increasing from four to eight patterns) and then point out where on the screen the pattern was initially shown (Sahakian et al., 1988). The test is terminated if a subject performs an error on three consecutive trials within a specific level (e.g., six patterns). The primary outcome variables for this test are the mean number of errors made before a stage was successfully completed as well as the number of stages completed on the first trial. The resultant value is calculated by summing the total errors for all attempted stages and dividing the result by the number of successfully completed stages. A lower value is better. The number of stages completed on the first trial refers to the total number of stages within the test that were completed during the initial visualization of the stage. In this instance, a higher value is better.

#### Spatial span—SSP

The SSP test (Owen et al., 1990) is a computerized visuo-spatial analog of the Corsi Block Tapping Test (Milner, 1971), with the corresponding outcome measures assessing working memory capacity. In this test, white squares are shown on the computer screen, some of which change color in a variable sequence, ranging from 5 squares to 9 squares as the complexity of the test increases. The subject is required to touch the boxes that changed color in the same order that they were displayed by the computer (Smith et al., 2013). The test is terminated if a subject performs at least one error on three consecutive trials within a specific level (e.g., 8 squares). The primary outcomes of this test are span length and latency. Span length refers to the longest sequence successfully recalled by the participant. The participant has three attempts at each level, with the maximum score possible being nine. A higher value is better. Latency refers to the amount of time taken to initiate the first response of the span length, and is measured from the end of the presentation phase until the participant touches the screen. A lower latency value is better.

### Physiological and subjective measurements

Core temperature (t_core_) and skin temperature (t_sk_) were recorded continuously throughout testing. Skin temperature was recorded at four sites on the left side of the body; mid-chest, thigh, upper arm, and calf (Payne et al., 1994), and mean skin temperature was calculated using the formula 0.3(*t*_chest_ + *t*_arm_) + 0.2(*t*_thigh_ + *t*_leg_) (Ramanathan, 1964). Both t_core_ and t_sk_ are reported as averages over the duration of each cognitive assessment. In addition, the dynamic change in t_core_ (Δ°C^.^h^−1^) was determined for each hour, as well as for the physical work portion of the protocol and during the cognitive assessment periods. Pre- and post- body mass was recorded to determine potential exercise-induced dehydration.

#### Heat stress index (HSI)

The HSI value represents the heat loss required to maintain a thermal steady state (*E*_req_) divided by the maximum potential for evaporation (*E*_max_) from the body through the TPC environment (McLellan et al., 2013). A value <1.0 on the HSI represents a compensable heat stress environment, whereas a value >1.0 reflects an uncompensable heat stress situation where the rate of heat storage will continue to increase and eventually cause physical and cognitive impairments (McLellan et al., 2013). The HSI of the HOT environment was determined using the following equations, which are applicable to individuals performing work/exercise in an encapsulated (e.g., wearing TPC) environment (McLellan et al., 2013):

$$\begin{array}{l} E_{\text{req}}=\left(\text{M}-{\text{W}}_{\text{ex}}\right)+{\text{A}}_{\text{D}}{\;}^{.}\;\left({\text{T}}_{\text{a}}-{\text{T}}_{\text{sk}}\right){\;}^{.}\;I_{\text{T}}^{-1} \end{array}$$

Where M is the rate of heat production, W_ex_ is the external work from the transfer of some of the total energy produced, A_D_ is the Dubois surface area (m^2^), *I*_T_ is the total thermal clothing insulation (m^2^.°C.W^−1^), T_sk_ is the mean skin temperature, and T_a_ is the ambient temperature (McLellan et al., 2013). Due to participants performing the cognitive testing battery while seated, metabolic heat production was estimated using a relative oxygen consumption of 3.5 mL.kg.min^−1^ (0.311 L.min^−1^ for the current study) and converting it to Watts (1 L O_2_ = 335 W) resulting in an estimated metabolic heat production (assuming respiratory exchange ratio = 0.85) of 104.4 W.

$$\begin{array}{l} {\text{E}}_{\text{max}}=16.5\cdot i_{\text{m}}\cdot I_{\text{T}}^{-1}\cdot {\text{A}}_{\text{D}}\cdot \left({\text{P}}_{\text{sk}}-\phi_{\text{a}}\cdot {\text{P}}_{\text{a}}\right) \end{array}$$

Where 16.5 is the Lewis Relation (°C. kPa^−1^), *i*_m_ is the Woodcock vapor permeability coefficient, P_sk_ is the skin saturation vapor pressure, ϕ_a_ is the ambient relative humidity, and P_a_ is the ambient saturation vapor pressure (McLellan et al., 2013). The precise insulation factor of the CFA wildland firefighting TPC was not known, so a conservative value from structural firefighting TPC of 1.55 clo (0.240 m^2.°^C^−1.^W^−1^) and a Woodcock vapor permeability coefficient of 0.27 (Selkirk and McLellan, 2004) was utilized to approximate HSI in the present study. The HSI data are presented in the Discussion.

### Statistical analysis

Normality for each of the dependent variables was confirmed using the Shapiro-Wilks test, while outliers were evaluated using box-plots and Cook's distance values. A within-subject analysis of variance (ANOVA) was conducted on t_core_, mean t_sk_, and change in body mass, whereas a within-subject analysis of co-variance (ANCOVA) was conducted for cognitive function performance recorded over time. Two subjects were unable to complete the 3-h protocol in the HOT, and one additional subject did not complete cog 4, due to experiencing minor heat illness symptoms; however, all physiological and cognitive measures are included in the analysis up to their withdrawal from testing. In both ANOVA and ANCOVAs, condition (HOT, CON) and time were entered into the model as fixed factors and subject was entered as a random effect to control for randomly missing data. Main effects (condition or time) and interaction effects (condition^*^time) were determined. If the ANOVA or ANCOVA revealed a significant interaction effect, simple effects analysis was conducted using Bonferroni correction to determine any differences between the estimated marginal means. Due to the self-paced nature of the rakehoe task, the amount of work completed during each hour of the protocol was factored into the cognitive function analyses as a covariate. Physical performance data have been previously reported (Larsen et al., 2015). Partial eta-squared (η^2^_*p*_) was calculated for the dependent variables as a measure of effect size, with values for small, medium, and large effect sizes set at 0.01, 0.06, and 0.14, respectively (Fritz et al., 2012). All statistical analyses were performed using IBM SPSS Statistics V. 21.0 (IBM Corp., Armonk, NY) with an alpha level of 0.05 set for all statistical procedures. All data are represented as adjusted means ± standard error of the mean (SEM) based on the estimates with rakehoe work performance as a covariate in the model.

## Results

### Subject characteristics

There were no differences in mass (PRE: 89.2 ± 2.8 kg vs. 89.2 ± 2.8 kg; POST: 89.1 ± 2.8 kg vs. 89.7 ± 2.8 kg), body mass index (BMI; 27.5 ± 1.0 kg^.^m^−2^ vs. 27.5 ± 1.0 kg^.^m^−2^), or percent change in body mass (−0.1 ± 0.3% vs. 0.5 ± 0.3%) for the CON and HOT trials, respectively (*p* > 0.05).

### Skin and core temperatures

**Figure 1:**
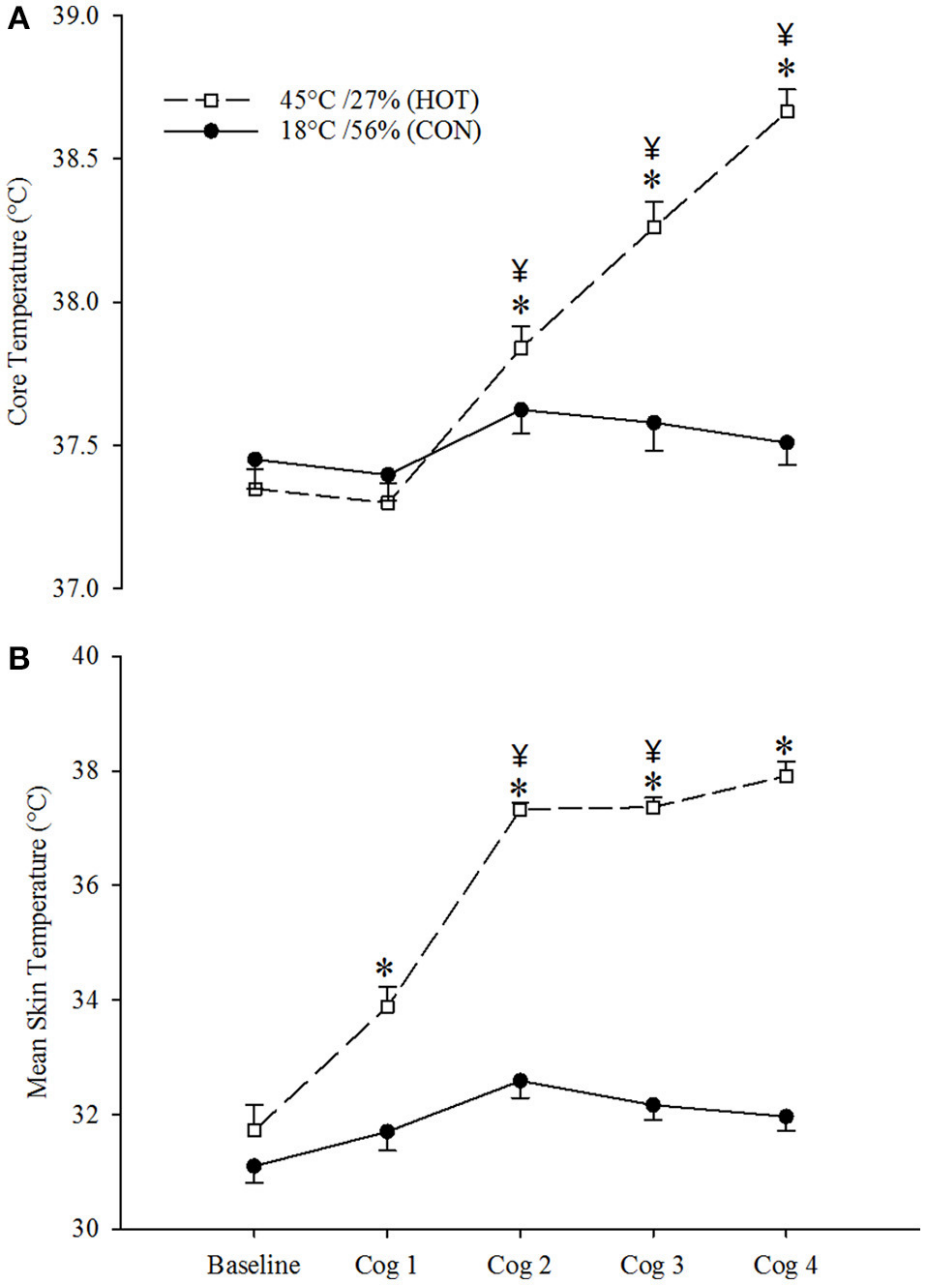
Average core **(A)** and skin **(B)** temperatures at each cognitive testing time point during the CON (open circles) and HOT (closed circles) conditions. Data are mean ± SEM. ^*^indicates different from CON (*p* < 0.001). ^¥^indicates different from Baseline within-condition for HOT (*p* < 0.0001) and CON (*p* < 0.01).

Mean t_sk_ and t_core_ values during the 15-min for each of the cognitive testing trials are shown in Figure 1 Data for t_core_ revealed a significant interaction effect [*F*_(4, 66)_ = 32.628; *p* < 0.001, η^2^_*p*_ = 0.664]. Simple effects analysis revealed no difference between CON and HOT at baseline and cog 1; however, there was an increase in core temperature during HOT of 0.3 ± 0.1, 0.7 ± 0.1, and 1.2 ± 0.2°C at cog 2, cog 3, and cog 4, respectively, when compared to CON (*p* < 0.01; η^2^_*p*_ = 0.128, 0.494, 0.679, respectively). Within-conditions, there was no difference in core temperature compared to baseline in CON, whereas HOT revealed significant differences at all cog trials (*p* < 0.0001; η^2^_*p*_ = 0.813). Data for t_sk_ also revealed a significant interaction effect [*F*_(4, 74)_ = 48.953; *p* < 0.001, η^2^_*p*_ = 0.726]. Simple effects analysis revealed no difference in t_sk_ at baseline between HOT and CON; however, t_sk_ for HOT was 2.1 ± 0.2, 4.8 ± 0.3, 5.1 ± 0.3, and 5.6 ± 0.3°C higher than CON for cog 1, cog 2, cog 3, and cog 4, respectively (*p* < 0.001; η^2^_*p*_ = 0.379, 0.748, 0.782, 0.777, respectively). Within-conditions, t_sk_ at cog 2 and cog 3 were significantly greater than baseline in CON (*p* < 0.01; η^2^_*p*_ = 0.263) while t_sk_ at all cog trials were elevated from baseline in HOT (*p* < 0.0001; η^2^_*p*_ = 0.882).

Dynamic change in t_core_ for the baseline cognitive assessment (cog 1), the mean of the 3-h experimental protocol (expressed as °C^.^h^−1^ for the mean of the six work bouts), and the mean during the three cognitive assessment trials (cog 2, 3, and 4) are depicted in Table 1. The mean change in core temperature over the entire 3-h protocol increased significantly faster in the HOT compared to fundamentally no change in the CON condition (*P* < 0.001). However, the change in core temperature during the cognitive assessment trials was falling in both conditions, with the rate of decrease being larger in CON compared to HOT (*P* < 0.001).

**Table 1:** Dynamic change in t_core_ (in °C.hr^−1^) for mean change over the entire experimental protocol (average), mean for all physical work portions (exercise), and mean for all cognitive assessment trials (cognitive).

	**Baseline (°C^.^hr^−1^)**	**Entire protocol (°C^.^hr^−1^)**		
**Condition**	**Average**	**Average**	**Exercise**	**Cognitive**
CON	−0.08 ± 0.1	0.01 ± 0.03	0.53 ± 0.14	−0.63 ± 0.12
HOT	−0.09 ± 0.1	0.43 ± 0.04^*^	0.76 ± 0.20	−0.15 ± 0.20^*^

Data are mean ± SEM.

* *indicates different from the CON condition (p < 0.05)*.

### Cognitive function

**Figure 2:**
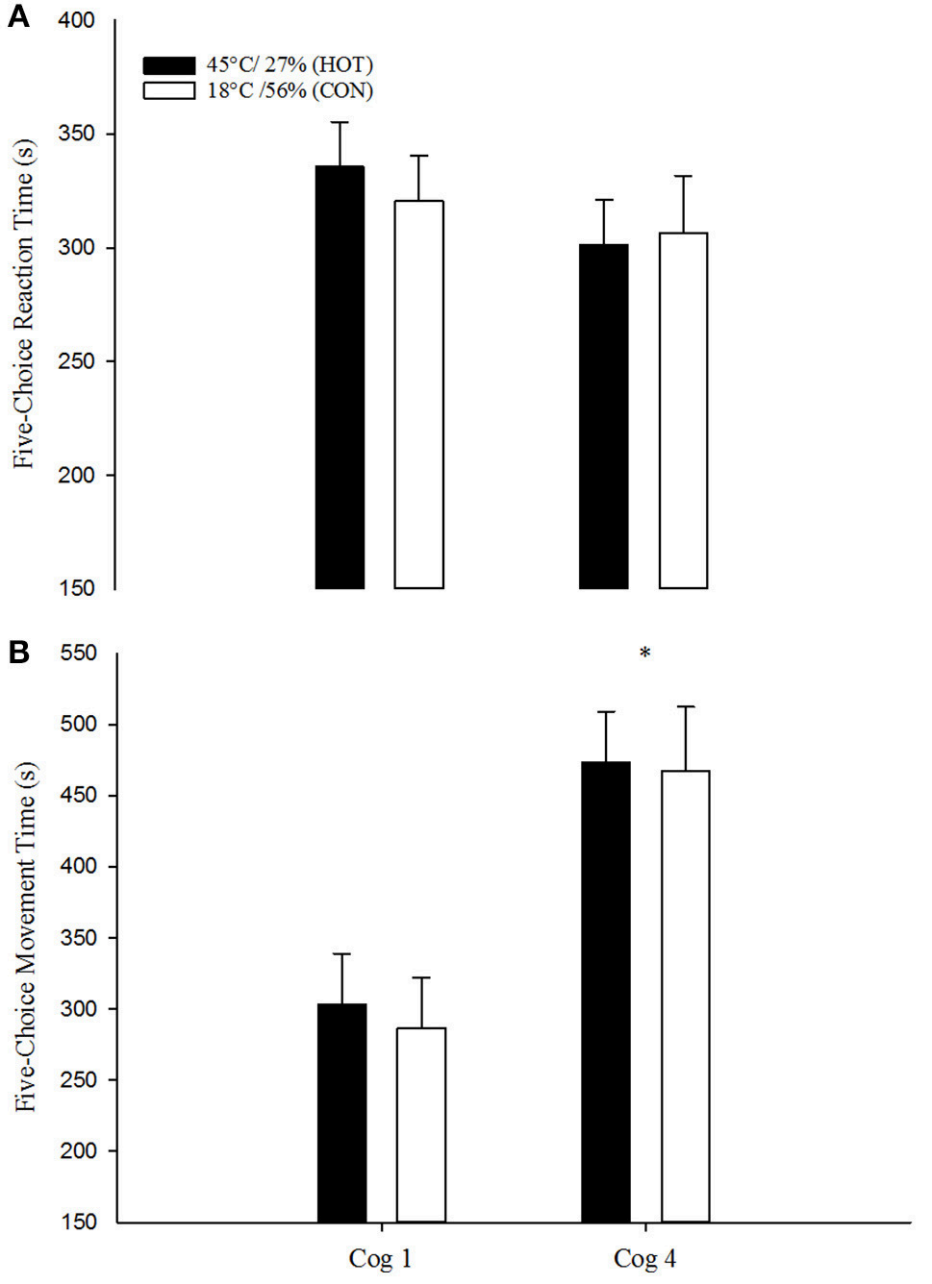
Mean reaction time **(A)** and movement time **(B)** for five-choice reaction time (RTI) in the CON (open boxex) and HOT (closed boxes) conditions. Data are mean ± SEM. ^*^Significant time effect from cog 1 within-conditions CON and HOT (*p* < 0.05).

**Figure 3:**
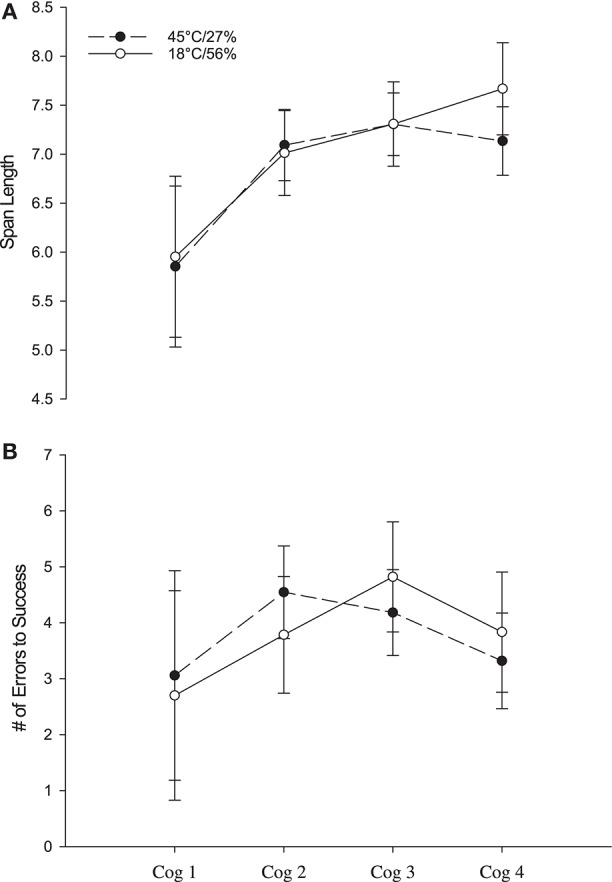
Mean spatial span length **(A)** on the spatial span (SSP) test and mean number of errors to success on the last level **(B)** for the paired associates learning (PAL) test in CON (open circles) and HOT (closed circles). Data are mean ± SEM.

Dependent measures for the three cognitive tasks are reported in Figures 2, 3. Five-choice movement time (Figure 2A) revealed a significant main effect for time [*F*_(1, 23)_ = 5.752; *p* = 0.03, η^2^_*p*_ = 0.200] but not condition [*F*_(1, 23)_ = 0.71; *p* = 0.41, η^2^_*p*_ = 0.030] or the interaction [*F*_(1, 23)_ = 0.14; *p* = 0.71, η^2^_*p*_ = 0.006]. Span length (Figure 3A) on the SSP test revealed no significant main effects for condition (*F*_1, 10.9_ = 0.594; *p* = 0.46, η^2^_*p*_ = 0.052) or time [*F*_(1, 11.6)_ = 2.211; *p* > 0.14, η^2^_*p*_ = 0.364], and no interaction effect [*F*_(3, 59)_ = 0.397; *p* = 0.76, η^2^_*p*_ = 0.020]. Latency on the SSP test revealed no main effect for condition [*F*_(1, 8.5)_ = 2.425; *p* = 0.16, η^2^_*p*_ = 0.222], time [*F*_(3, 8.9)_ = 2.776; *p* = 0.10, η^2^_*p*_ = 0.482], and no interaction effect [*F*_(3, 59)_ = 0.537; *p* = 0.54, η^2^_*p*_ = 0.027]. Similarly, for the PAL test, the mean number of errors (Figure 3B) required for completion of a successful level showed no significant main effects for condition [*F*_(1, 56)_ = 0.000; *p* = 0.989; η^2^_*p*_ = 0.000], time [*F*_(3, 56)_ = 0.70; *p* = 0.56, η^2^_*p*_ = 0.036] or the interaction [*F*_(3, 56)_ = 0.499; *p* = 0.68, η^2^_*p*_ = 0.026]. The number of levels completed on the first trial also revealed no significant main effects for condition [*F*_(1, 59)_ = 0.155; *p* = 0.70, η^2^_*p*_ = 0.003], time [*F*_(3, 59)_ = 0.947; *p* = 0.42, η^2^_*p*_ = 0.046] or the interaction [*F*_(3, 59)_ = 1.209; *p* = 0.31, η^2^_*p*_ = 0.058].

## Discussion

This study examined the effects of performing simulated wildland firefighting work in a very hot environment on cognitive function. Although attaining a moderate level of hyperthermia, the cognitive performance measures were unaffected by work in the heat. This preservation of cognitive function appears to be related to the negative dynamic t_core_ change during the cognitive assessment trials, despite the elevation in absolute t_core_ measured at the end of the protocol. Furthermore, through *ad libitum* fluid intake, firefighters in the present study were able to maintain their body mass to avoid dehydration, which may have assisted in the maintenance of cognitive performance.

This research builds upon the existing literature, which has predominantly utilized simple cognitive tasks, by incorporating cognitive tests with increasing levels of complexity during the experimental protocol. The two cognitive tests assessing working memory and planning (SSP) and visual memory (PAL) revealed no significant differences in performance between conditions (CON vs. HOT) or across the 3-h protocol. The lack of impairment in cognitive function was observed despite moderate levels of hyperthermia experienced by the firefighters, as evidenced by an average t_core_ of 38.7°C during the final cognitive assessment in the HOT trial. This is a similar level of thermal strain that has been imposed by other studies examining the effects of environmental and metabolic heat stress on various cognitive components (Aoyagi et al., 1996; Gonzalez et al., 1997; Smith and Petruzzello, 1998; Selkirk and McLellan, 2004; Morley et al., 2012). The current findings support previous research that reported no changes in short-term memory, sustained or divided attention, and reaction time following 50-min of continuous treadmill exercise in a 30–33°C environment (Morley et al., 2012). In addition, Rayson et al. (2005) found no change in rapid visual information processing, spatial memory span, and choice reaction time following a firefighting simulation; however, the cognitive assessment was performed 30-min post-simulation, potentially eliminating any adverse effects that may have occurred immediately following the simulation. Generally, decrements in cognitive function have been observed in studies that also report hypohydration or dehydration of >2% body mass loss (Gopinathan et al., 1988; Cian et al., 2001; Lieberman et al., 2005). Notably, the *ad libitum* water consumption during the current study maintained body mass at pre-exercise levels (PRE: 89.1 ± 2.8 kg vs. POST: 89.7 ± 2.8 kg), and therefore did not reach the 2% loss required to elicit concentration and working memory decrements (Sharma et al., 1986; Cian et al., 2001). This preservation of euhydration appears to have assisted in preventing impairments to cognitive function.

There are several other possible explanations as to why cognitive function was not impaired in the present study following moderate hyperthermia. Previous research has shown that it is not the absolute t_core_ reached that influences cognitive function, but the rate at which t_core_ changes (Hancock and Vasmatzidis, 2003) as well as the direction of the change (Allan and Gibson, 1979; Allan et al., 1979; Gibson and Allan, 1979; Gibson et al., 1980). The rates of increase in t_core_ that have been reported to result in decrements for vigilance, dual task, tracking, and simple mental performance are 0.06, 0.22, 0.88, and 1.33°C^.^h^−1^, respectively (Hancock and Vasmatzidis, 2003). Although the average rate of t_core_ increase was 0.43°C^.^h^−1^ (mean over the 3-h protocol) and 0.76°C^.^h^−1^ (mean for the exercise portions of the protocol) in the HOT condition, the direction of t_core_ change during the cognitive function assessments were negative for both the CON and HOT conditions (Table 1). It is possible that the drop in t_core_, and the concurrent maintenance of cognitive performance between physical work bouts, was facilitated by firefighters removing their helmets and opening their jackets during this time (as is standard practice for firefighters during rest breaks). This behavioral adaptation to the environmental condition, in conjunction with the maintenance of euhydration, most likely accounted for the lack of cognitive impairments observed.

The negative rate of dynamic t_core_ change during the cognitive assessment trials, despite the severe environmental conditions imposed, may be explained using the HSI (Gonzalez and Sawka, 1988). When calculating HSI using the method described by McLellan et al. (2013), the HSI value while wearing TPC in a resting condition (as was the case during the cognitive assessments) at 45°C and 27% humidity is 0.85. It should be noted that the total clothing insulation factor for wildland firefighting would likely be even lower, and the Woodcock vapor permeability coefficient higher, than structural firefighting TPC, thus resulting in an even lower HSI value. Furthermore, participants were allowed to remove their helmet and open their jackets during the rest periods, which would further decrease the HSI value and create a compensable heat stress environment during the cognitive assessments.

Five-choice movement time increased across the 3-h period during both trials, indicating that boredom may have been a cause rather than hyperthermia. It is possible that some of the tests used to assess cognitive function were too simple for the healthy volunteer wildland firefighters included in this study; it is well known that task complexity plays a major role in determining the effects of thermal stress on cognition (Hancock, 1982). Simple tasks, such as reaction time and mental transformation, are not as vulnerable to heat stress when compared to more complex tasks such as vigilance, tracking, and dual tasks (Grether, 1973; Hancock, 1981, 1982). However, the present study employed two cognitive tests (SSP and PAL) that increased in complexity with each subsequent level, which should have mitigated any issues arising from test simplicity. The use of repeated measures cognitive testing also raises the possibility of improvement due to practice effects, which could potentially mask decrements due to heat stress. However, a double familiarization session and a double baseline were utilized in the present study, which should have minimized the occurrence of learning effects. Finally, it is possible that the firefighters modulated their physical work output on the raking task in order to preserve cognitive function. In a study conducted by Mohr et al. (2012), soccer players were observed to cover less distance but to improve technical performance (i.e., passing/crossing success rates) during games in the heat when compared to temperate conditions. Although outside of the scope of the present research, future studies should therefore investigate pacing strategies in firefighting personnel to better understand this potential trade-off between physical and cognitive performance.

The protocol used in the current study represents a significant step forward toward a high fidelity simulation of fireground work. Direct application of the current findings to wildfire suppression environments should, however, be made cautiously. Firefighters face multiple stressors during suppression (Aisbett et al., 2012), and it is likely that the complexity of the fireground was not precisely replicated in the current protocol. An obvious next step is to assess cognitive function in wildland firefighters as they concurrently perform physical work in hot conditions, in order to maintain an increased t_core_ and more realistically reflect working conditions. Another possible avenue for future research is to explore the impact of wind speed on firefighters' thermal physiology and cognition. Increased wind speed will create an environment for elevated heat loss through evaporation (Saunders et al., 2005), which would likely decrease core temperature and further protect cognitive function. Finally, exploration of the stressors (e.g., wind speed, ambient heat, radiation) in tandem would provide more nuanced information to fire agencies regarding the health and wellbeing of their firefighters.

## Conclusions

All cognitive function assessments utilized in the current study, including those with increasing levels of complexity, were unimpaired when performing 3 h of intermittent, simulated firefighting tasks in 45°C. The maintenance of firefighters' cognitive function in the current study is likely attributed to: (a) their euhydration throughout testing (as indicated by the maintenance of pre-trial body mass), and; (b) a relative drop in core temperature when the cognitive assessments were performed. These data should compel fire authorities to continue to provide free access to fluid during wildfire deployments and, wherever possible, allow intermittent rest breaks between physical efforts. More research is required to explore the operational consequences for workers who have to perform concurrent physical and cognitive work tasks in very hot environments. The impacts of multiple stressors (e.g., very hot temperatures and high winds) on thermoregulation and cognition should also be a research priority.

## Ethics statement

Ethical approval was obtained from the Deakin University Human Ethics Committee, and written informed consent was attained prior to each subjects' first visit to the laboratory.

## Author contributions

FW: data acquisition and analysis, and manuscript writing. BA: study conception and manuscript editing. BM: data interpretation and manuscript editing. BL: study design, data acquisition, and manuscript writing.

### Conflict of interest statement

The authors declare that the research was conducted in the absence of any commercial or financial relationships that could be construed as a potential conflict of interest.
